# Rare primary pleomorphic rhabdomyosarcoma of the thyroid gland with lung and adrenal metastases: a case report and literature review

**DOI:** 10.3389/fonc.2025.1756395

**Published:** 2026-01-20

**Authors:** Rami Altabbouche, Jacek Musiał, Anna Jałocha-Kaczka, Łukasz Dziki, Kasper Maryńczak, Krzysztof Kaczka

**Affiliations:** 1 Faculty of Medicine, Medical University of Lodz, Łódź, Poland; 2 Department of Pathomorphology, Medical University of Lodz, Łódź, Poland; 3 Department of Otolaryngology, ENT Oncology, Audiology and Phoniatrics, Medical University of Lodz, Łódź, Poland; 4 Department of General and Oncological Surgery, Medical University of Lodz, Łódź, Poland

**Keywords:** adrenal metastases, endocrine surgery, immunohistochemistry, pleomorphic rhabdomyosarcoma, primary thyroid rhabdomyosarcoma, pulmonary metastases, rare endocrine malignancy, thyroid sarcoma

## Abstract

Primary thyroid pleomorphic rhabdomyosarcoma (PRMS) is an extremely rare and aggressive neoplasm, with only five cases reported in the literature to date. We present the sixth reported case of primary thyroid PRMS and the first case demonstrating distal metastases to the lungs and left adrenal gland in a 71-year-old female. The patient underwent total thyroidectomy with lymphadenectomy, with histopathological evaluation confirming PRMS and demonstrating a highly aggressive biological behavior. The immunophenotypic profile showed strong immunopositivity for skeletal muscle markers, including desmin and vimentin. Further computerized tomography imaging identified a metastasized lesion in the left adrenal gland and a diffuse lung nodular dissemination. The patient received a personalized treatment plan which combined both systemic chemotherapy and radiotherapy. After four cycles of chemotherapy, the patient achieved partial regression of metastatic lesions. The highly aggressive nature of PRMS emphasizes the importance of having individualized and multidisciplinary treatment approaches to such rare cases.

## 1 Introduction

Primary thyroid sarcoma is a rare malignancy which originates from the mesenchymal tissue of the thyroid gland and accounts for only 0.01% to 1.5% of all thyroid cancers (1). Among these, thyroid rhabdomyosarcoma (RMS) is an extremely rare subtype of primary thyroid cancers, with only five cases reported in the literature to date (2–6).

RMS, in general, are very uncommon in adults, representing fewer than 1% of all adult malignant tumors (7), but relatively more frequent in children, comprising 4% to 6% of pediatric malignancies (8). In this age group, approximatively 40% of cases are located in the head and neck regions (9), although thyroid gland involvement in children is exceptionally rare, with only two embryonal-type cases documented (2, 3). In adults, the pleomorphic variant predominates (6).

The rarity of this tumor in adults is particularly important for understanding its complex clinical presentation and management. To our knowledge, this is the sixth reported case of primary thyroid RMS, the fourth in an adult and the first to exhibit combined adrenal and pulmonary metastases.

## 2 Case report

### 2.1 Clinical presentation

A 71-year-old female patient was admitted with a history of severe dyspnea and an enlarged neck mass. The patient’s thyroid function test revealed no abnormalities; all its respective values were within normal ranges. The initial imaging modality performed in the patient was ultrasound sonography (USG). The USG showed no enlargements in the isthmus, and the right lobe of the thyroid gland had homogeneous parenchyma and typical echogenicity. In addition, the lower pole of the thyroid gland descended into the upper mediastinum. The left lobe of the thyroid was entirely nodular with a hypoechoic focal lesion in the deep region, displaying a non-parallel (taller-than-wide) orientation – a feature suggestive of malignancy. The biggest nodule assessed had a size equal to 38 × 34 × 40 mm and was classified as EU TIRADS PL5, which required an urgent endocrinological consultation and a Fine Needle Aspiration Biopsy (FNAB). Medial to the lesion, around the isthmus, there were several hypoechoic lesions visible of sizes up to 10 × 6 mm, but their nature could not be clearly determined as they may have corresponded to lymph nodes or possible changes in the thyroid isthmus. The trachea was displaced with a clear right-sided deviation. Moreover, in the left thyroid lobe, several hyperechoic lesions were classified as EU TIRADS PL3 with a size of 20 × 21 × 12 mm. In the deep part of the left lobe, a single hyperechoic lesion measuring 7.5 × 6 × 8 mm was visible (EU TIRADS PL3). The surrounding lymph nodes were not enlarged and had normal echotextures. The patient was qualified for laryngoscopy and the results were unremarkable.

### 2.2 Biopsy results: description and immunohistochemistry results

The tumor from the left lobe displayed numerous lymphocytes with scattered histiocytes and few small and medium-sized clusters of cells showing macronucleosis and hyperchromatosis. The cells had large reddish nucleoli with clearly visible pathological mitotic figures; they raised suspicion of atypia.

Immunohistochemistry revealed that the tumor cells were strongly positive for muscular markers: desmin (100% of the cells), vimentin (100%), CD31+ (30%), and had a high proliferation index (Ki-67 85-90%). Meanwhile, epithelial and thyroid differentiation markers were negative: CD34-, thyroglobulin-, TTF-1-, SOX10-, SMA- (positive in few cells), CK7-, CD45-, CK5/6-, p63-, PAX 8-, CKAE1/AE3 (positive in single cells).

### 2.3 Post-surgical specimen assessment

A total thyroidectomy with a middle cervical lymphadenectomy was performed. During surgery, the left lobe was found to be significantly enlarged with evidence of infiltration. The internal jugular vein and vagus nerve were dissected due to the extent of mass invasion. In addition, the recurrent laryngeal nerve was also damaged during surgery due to neuroinvasion.

**Figure 1:**
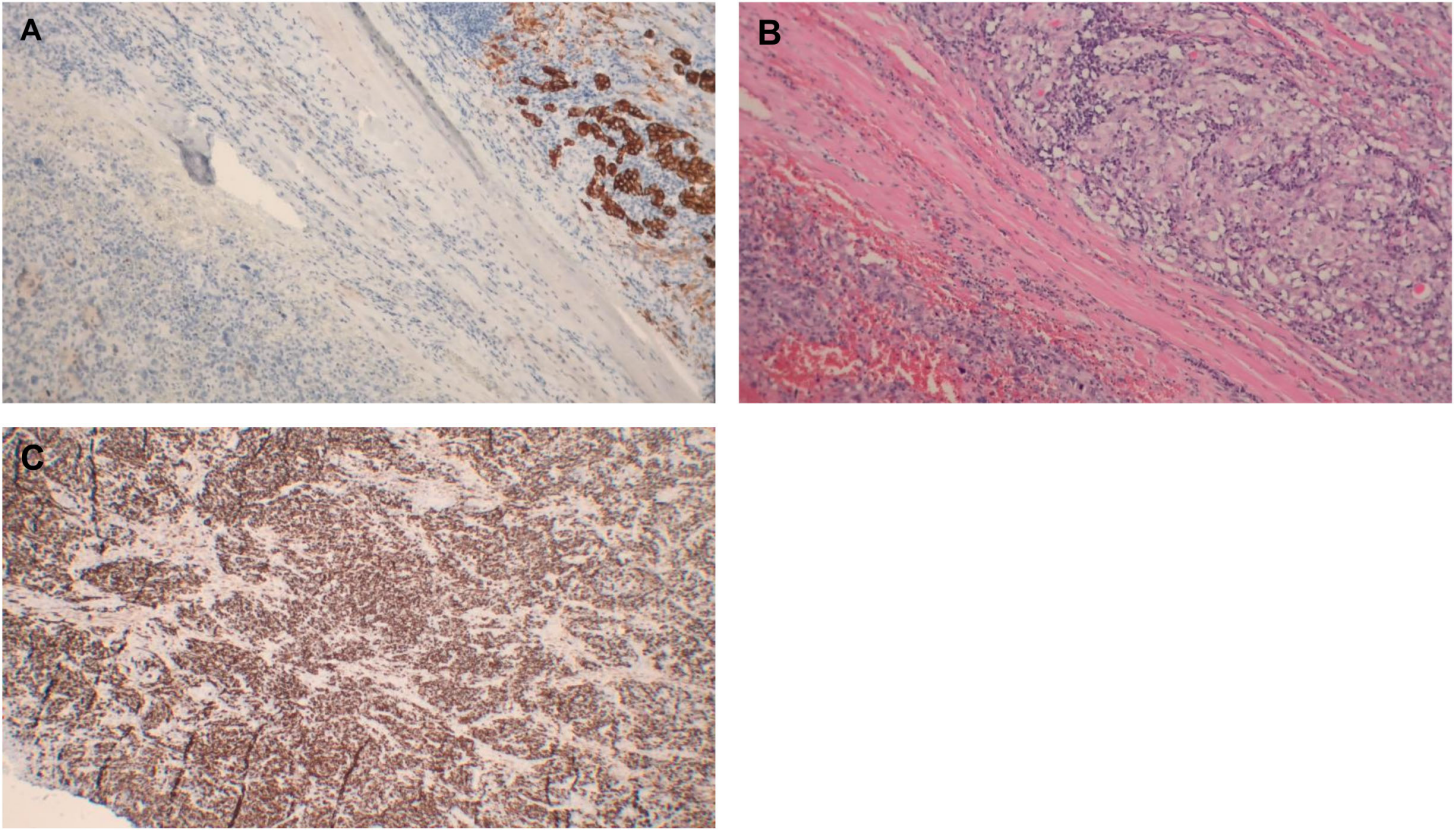
Immunohistochemistry results after thyroidectomy. **(A, B)** Normal thyroid tissue at 10× magnification: **(A)** Cytokeratin immunostaining highlights thyroid follicles; **(B)** Hematoxylin and eosin (H&E) staining shows preserved thyroid architecture. Thyroid follicles are absent. **(C)** Strong desmin expression at 5× magnification.

After the procedure, the microscopic appearance and immunophenotype of the thyroid malignancy confirmed the pleomorphic rhabdomyosarcoma (PRMS) diagnosis. The left thyroid lobe showed indeed a pleomorphic malignant neoplasm with areas of spindle cell morphology. Numerous large to very large cells were observed, characterized by intensely eosinophilic cytoplasms. The immunophenotype was mostly consistent with the FNAB results and displayed CKAE1/AE3+ in some cells, CD31+ in approximately 30% of cells, and the index of proliferation Ki-67 equal to 85-90%. The remaining markers were negative: CD34-, EMA- (positive in single cells), thyroglobulin-, and TTF-1-. Moreover, immunohistochemical staining showed that both desmin and vimentin were strongly positive in all cells, SOX10-, SMA- (positive in few cells), CK7-, CD45-, CK5/6-, p63-, and PAX8 (**Figure 1**). Numerous mitotic figures were present, including atypical ones. Areas of necrosis covered about 30% of the tumor tissue. Hemorrhages, fibrosis, and hyalinization were also visible, with the tumor infiltrating deeply into the thyroid capsule and the perithyroid soft tissues.

The maximum size of the largest tumor was 9.5 cm; both neuroinvasion and angioinvasion were recorded. The assessment of three lymph nodes revealed no metastases (0/3). The right thyroid lobe had no neoplastic changes.

### 2.4 Adrenal and lung invasion

**Figure 2:**
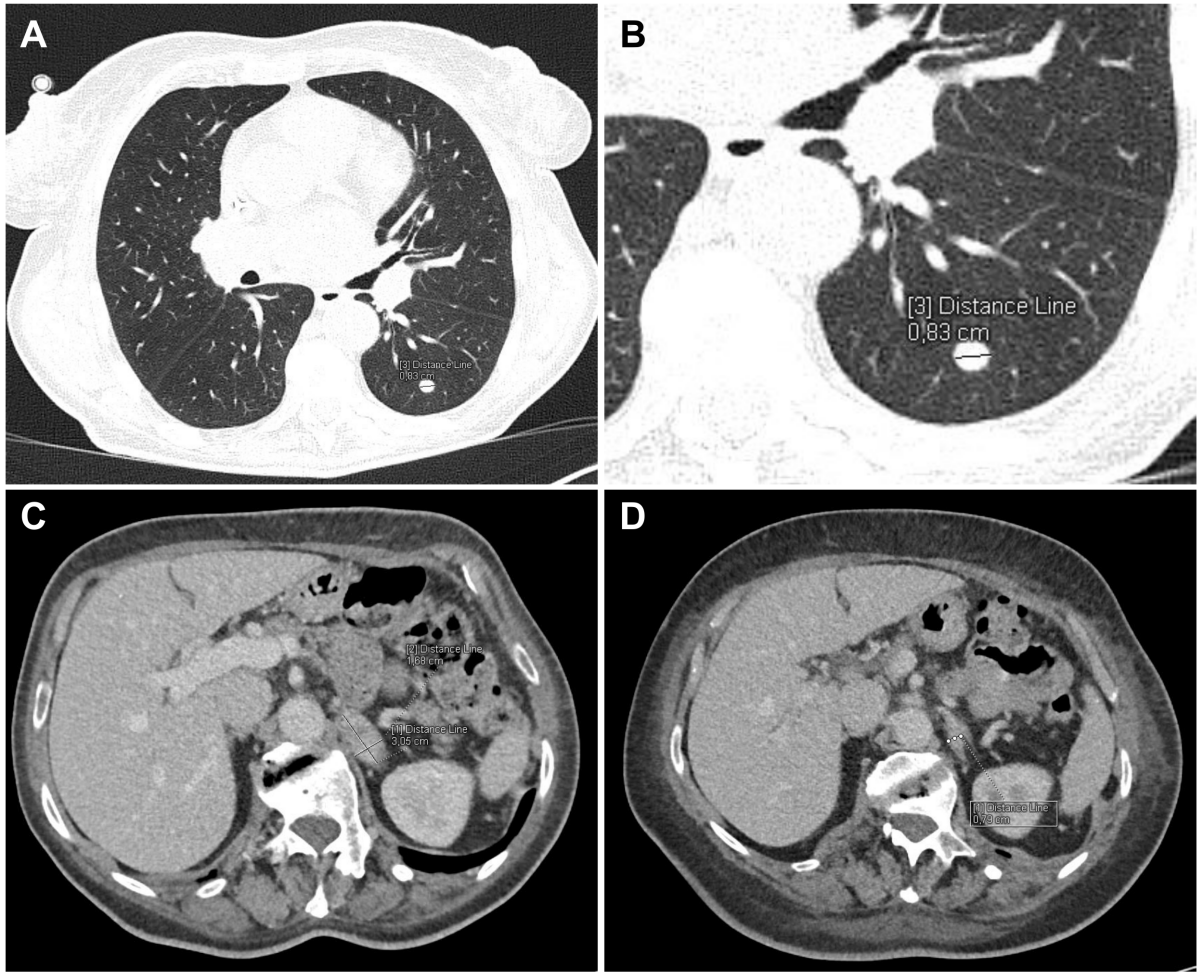
Contrast-enhanced CT of the thorax, abdomen, and pelvis. **(A, B)** CT scans of the lungs showing a well-circumscribed metastatic nodule (8 mm) in segment 6 of the left lung. **(C)** CT scan of the abdomen (venous phase) demonstrating a metastatic mass (30 × 17 mm) in the left adrenal gland. **(D)** CT scan of the abdomen performed 3 months earlier (venous phase) showing the left adrenal gland without metastatic changes.

Imaging studies revealed a metastasis to the left adrenal gland, with the lesion initially measuring 30 × 17 mm and subsequently decreasing to 15 × 15 mm following systemic treatment (**Figure 2**). Radiotherapy was administered to the surgical site. At first, the decision to perform left adrenalectomy was made based on initial imaging studies, which suggested a solitary lesion. However, it was later confirmed that this was not the only site of metastasis, as the malignant neoplasm had disseminated to both lungs, and the choice shifted towards palliative chemotherapy (**Figure 2**).

In addition, computerized tomography (CT) further demonstrated variable pulmonary involvement and a radiologic response after systemic therapy. An earlier CT showed radiological signs of progression with single nodules in the lower lobes of both lungs, measuring up to 15 mm, consistent with metastases. Subsequent CT scans between April and July 2025 showed a partial response according to RECIST 1.1 criteria, with a decrease in the number and size of pulmonary nodules. Specifically, a 5-mm nodule in segment P3, an 8-mm nodule in segment L6, and a 4-mm subpleural nodule in segment L9 were detected. In the location of the largest nodule in segment L8, a difficult-to-measure area of peripheral consolidation was visible, confluent with atelectatic changes and subparallel pleural thickening. The pleura of the lower lobes was irregularly thickened without visible postcontrast enhancement (up to 6 mm on the right and up to 4 mm on the left, respectively). In segment P10, a subpleural nodule decreased to 5 mm and partially merged with the new pleural thickening. The area of peripheral parenchymal consolidation in segment P6 had slightly enlarged and was considered more consistent with an atelectatic change.

### 2.5 Treatment plan

Following thyroidectomy (R1 procedure), the 71-year-old patient was initially treated with doxorubicin and ifosfamide from January 2025 to April 2025 (**Table 1**). During the first cycle, she developed febrile neutropenia, thrombocytopenia, and anemia. In January 2025, she experienced significant extravasation of doxorubicin in the right antecubital fossa, which was managed with DMSO and cold compresses without subsequent ulceration or blister formation. Right cubital vein thrombosis was also diagnosed and treated with low molecular weight heparin (enoxaparin). Primary prophylaxis against febrile neutropenia was provided using G-CSF. Supportive treatment relieved the symptoms.

**Table 1:** Summary of all chemotherapeutic agents and cycles used for PRMS treatment.

Cycle 1	doxorubicin and ifosfamide with symptomatic medications and G-CSF
Cycle 2	docetaxel and gemcitabine with symptomatic medications and G-CSF
Cycle 3	gemcitabine and docetaxel in two doses with good tolerance and G-CSF
Cycle 4	gemcitabine alone with good tolerance

After the regimen completion, the patient was transitioned to second-line chemotherapy with gemcitabine and docetaxel, starting in May 2025. The first dose of the third cycle was administered at the end of May 2025, with good immediate tolerance. The last cycle included only gemcitabine, and the patient had no complications.

Throughout the treatment, supportive measures effectively reduced lower limb edema, while respiratory infections were managed with empirical antibiotics (amoxicillin with clavulanic acid and clarithromycin). The patient was discharged home in good general condition after each cycle with recommendations for continued treatment and supportive care.

## 3 Discussion

The treatment and diagnosis of thyroid PRMS have been poorly discussed in international guidelines. Our case report represents the fourth documented case of PRMS in adults and the sixth reported case of primary thyroid RMS. Thyroid sarcomas are highly aggressive (5) and present multiple challenges in both treatment and diagnosis due to their rarity. Adult RMS, in particular, has an aggressive clinical course, with a 5-year survival rate of merely 35% in adults (10). In our case, an urgent total thyroidectomy with lymphadenectomy was performed due to the extreme mass effect of the 9.5-cm tumor in the left thyroid lobe. Following surgery, further investigations with a CT scan revealed metastases to the left adrenal gland and the lungs, prompting an adjuvant chemotherapeutic regimen. The patient received four cycles of systemic chemotherapy with doxorubicin and ifosfamide, followed by gemcitabine and docetaxel. Additionally, the patient underwent four cycles of radiotherapy to the surgical site.

Due to its rarity, molecular heterogeneity, and variable chemosensitivity, the management of adult RMS is complex when compared with the pediatric form. Most treatment regimens for adult RMS are guided by studies conducted in pediatric populations, in which standardized protocols have been more thoroughly established (11). Pediatric treatment strategies are typically based on combinations of ifosfamide, vincristine, and actinomycin, with or without metronomic cyclophosphamide for maintenance; by contrast, adult sarcoma management more commonly incorporates single-agent doxorubicin or ifosfamide–epirubicin (11, 12).

At the biological level, molecular and pharmacological analyses indicate that adult RMS often display variable responses to systemic therapy compared with pediatric counterparts (13). As a result, therapeutic approaches differ according to sarcoma subtype, ranging from relatively chemosensitive embryonal and alveolar RMS to the poorer chemosensitivity characteristic of the pleomorphic variant (12). The complexity of management lies not only in subgroup classification but also in significant intertumoral variability in signaling pathways, drug sensitivity, and resistance mechanisms, which further complicates therapeutic strategies in adult RMS (13, 14). In keeping with these observations, pleomorphic RMS is frequently associated with highly complex karyotypes, unlike the more defined karyotypic patterns observed in alveolar and embryonal RMS (12). For localized disease, surgical resection is the first-line treatment to limit tumor spread (13). When indicated, postoperative radiotherapy administered as adjuvant therapy has demonstrated improvements in overall survival and reductions in both local and distant disease spread compared with surgery alone (15).

Management becomes even more complex in the metastatic setting, particularly in PRMS, where optimal systemic therapy is not well defined. Although treatment strategies for metastatic disease remain debated (13), previously reported approaches have commonly included anthracycline- and ifosfamide-based chemotherapy, as well as gemcitabine- and taxane-containing combinations in advanced disease, despite limited high-level evidence of effectiveness (13). In the present case, an individualized, multimodal strategy was chosen due to the tumor’s high potential for rapid progression, with surgery followed by radiotherapy contributing to disease control.

Among five previously reported cases, none exhibited distant metastases. In the case reported by Febrero et al., the mass of the right hemi thyroid spread locally to the thoracic region, causing leftward tracheal deviation. The patient underwent surgery and biopsy, but eventually died 48 hours after the procedure due to cardiac insufficiency (5). Other cases had better prognoses with no further disease progression. For instance, two cases of thyroid sarcoma in children were treated successfully with lobectomy and chemotherapy without complications (2, 3). In adults, Ozaslan et al. reported a case in which total thyroidectomy alone contained the disease. Meanwhile, in the case of Alsugair et al., the use of a combination of four courses of chemotherapy (cisplatin and doxorubicin) and external radiotherapy helped regress the neck mass effectively before a total thyroidectomy (**Table 2**).

**Table 2:** Comparative summary of reported primary rhabdomyosarcoma cases of the thyroid gland, adapted from previously published case reports (2-6) and the current case.

Case	Age & sex	Type	Diagnostic imaging modality	Size (in cm)	Immunohistochemical profile	Distal metastasis	Treatment plan	Outcome
Furze et al. (2)	7 months old, Male	embryonal rhabdomyosarcoma	CT, MRI	5 x 4 x 4	non-diagnostic results	no	left hemithyroidectomy followed by chemotherapy (vincristine, actinomycin-D, and cyclophosphomide)	complete remission
Dutta et al. (3)	7 years old, Male	embryonal rhabdomyosarcoma	CT	6.5 x 5 x 4.5	desmin+, myogenin+, cytokeratin-, CD34-, CD31-	no	right hemithyroidectomy followed by six cycles of chemotherapy (doxorubicin, ifosfamide, mesna) and radiotherapy	complete remission
Ozaslan et al. (4)	68 years old, Male	primary pleomorphic rhabdomyosarcoma	USG, CT	5.5 x 4 x 3 on USG, 5 in diameter post-surgery	desmin+, vimentin+, Ki-67 (40%), CD38-, CD34-, BCL-2-, thyroglobulin-, pan-keratin-, pancreatin-, LCA-, SMA-, S100-, CD117-, AE1-AE3-, actin-, TTF-1-	no	total thyroidectomy	no recurrence, surveillance only
Febrero et al. (5)	67 years old, Male	primary rhabdomyosarcoma	USG, CT, PET	6	desmin+, vimentin+, actin+, myogenin+, AE1-AE3-, cam5-, EMA-, CEA-, TTF-1-, CD45 ALC-, CD34-, melan-A-, S-100-, CD31-, and CD68-	no	surgery	death 48 hours after surgery due to cardiac insufficiency
Alsugair et al. (6)	61 years old, Female	primary pleomorphic rhabdomyosarcoma	USG, CT and PET	4.6	desmin+, MyoD1, myogenin+, Ki-67 (40%), AE1-AE3-, EMA-, CEA-, TTF-1-, PAX8-, thyroglobulin-	no	total thyroidectomy, four cycles of neoadjuvant chemotherapy (cisplatin and doxorubicin), and radiotherapy	no recurrence
Current study (2025)	71 years old, Female	primary pleomorphic rhabdomyosarcoma	USG, CT	9.5	desmin+ (100%), vimentin+ (100%), CKAE1/AE3- (+ in single cells), EMA- (+ in single cells), CD31+ (30%), Ki-67 (85-90%), CD34-, thyroglobulin-, TTF-1-, SOX10-, SMA- (+ in few cells), CK7-, CD45-, CK5/6-, p63-, PAX 8-	left adrenal gland with nodular infiltration of the lungs	total thyroidectomy, radiotherapy and four cycles of chemotherapy (doxorubicin. ifosfamide, gemcitabine, docetaxel)	partial remission

A differential diagnosis was essential to rule out the possibility of other types of malignancies. To begin with, using immunohistochemistry, all cytokeratin, epithelial, and thyroid differentiation markers were negative, excluding the chance of undifferentiated carcinoma. Furthermore, the strong positivity of muscle markers in our samples helped us eliminate other types of sarcomas, such as sarcomas of myxoid origin, which were considered the second differential diagnosis. Additionally, the markers of synovial sarcoma (CK7, EMA) were negative, further refining the diagnosis.

The differences between adult and pediatric RMS lie within their biological heterogeneity, not only in clinical behavior but also at the molecular level. In pediatric RMS, particularly the alveolar subtype, canonical fusion oncogenes such as PAX3–FOXO1 and PAX7–FOXO1 act as molecular drivers and are used for both diagnostic and prognostic purposes, reflecting a comparatively clearer genetic background with recurrent translocations that influence tumorigenesis (16, 17). In contrast, adult RMS, including pleomorphic variants, often lack these characteristic pediatric fusion events and instead display a wider spectrum of molecular alterations (13, 14). Molecular studies have identified rare and novel fusion transcripts in adult RMS, including the RAB3IP–HMGA2 fusion reported in a head and neck RMS, emphasizing the potential importance of molecular profiling even in atypical presentations (16). Similarly, preclinical work has demonstrated that distinct signaling drivers, such as activating FGFR4 mutations, may influence RMS growth and response to targeted therapies (18). Although molecular testing was not performed in the present case, recognition of this genetic heterogeneity supports an individualized diagnostic and therapeutic approach in rare adult RMS occurring at uncommon anatomical sites such as the thyroid gland.

## 4 Conclusion

According to the literature, the two primary RMS of the thyroid gland in pediatric cases were of embryonal origin (2, 3), while they were exclusively classified as PRMS in adults. We report a rare case of PRMS of the thyroid gland confirmed by strong immunopositivity for desmin and vimentin. Our patient represents the sixth documented case worldwide and the first with both adrenal and pulmonary metastases, which made the treatment approach more personalized. The patient underwent total thyroidectomy followed by four cycles of combination chemotherapy and four courses of radiotherapy, achieving partial regression of metastatic disease. This case portrays the highly aggressive biological behavior of thyroid RMS and underlines the importance of immunohistochemical evaluation in its differentiation from other thyroid neoplasms. Continued reporting of similar cases is crucial to improving understanding of its clinical course and guiding the development of specific therapeutic strategies.

## Data Availability

The original contributions presented in the study are included in the article/supplementary material. Further inquiries can be directed to the corresponding author.
